# An Unexpected Turn of Events in a Patient With Mitral Valve Endocarditis

**DOI:** 10.7759/cureus.42121

**Published:** 2023-07-19

**Authors:** Chelsea-Jane Arcalas, Richard Artola, Megan E Hanna, Han Li, Sydney Levy, Gautam S Kalyatanda

**Affiliations:** 1 College of Medicine, University of Florida, Gainesville, USA; 2 Internal Medicine, University of Florida College of Medicine, Gainesville, USA; 3 Infectious Diseases and Global Medicine, University of Florida College of Medicine, Gainesville, USA

**Keywords:** daptomycin-related adverse effect, prosthetic joint infection, daptomycin, covid-19, daptomycin-induced acute eosinophilic pneumonia

## Abstract

A variety of gram-positive infections can be treated with daptomycin. Daptomycin-induced acute eosinophilic pneumonia (DEP) is a rare adverse drug reaction with nonspecific clinical findings of dyspnea, dry cough, and fever. Although diagnostic criteria exist, prompt recognition is important to prevent rapid progression and respiratory failure. In this case, a 69-year-old female was initially admitted due to a prosthetic joint infection; however, her case was complicated by DEP.

## Introduction

Daptomycin is a cyclic lipopeptide bactericidal antibiotic initially approved for its activity against gram-positive organisms including methicillin-resistant *Staphylococcus aureus* (MRSA) and vancomycin-resistant *Enterococci *(VRE) [1]. It can be used for infections of the skin, soft tissue, and bone, as well as bacteremia and infective endocarditis. It has a variety of off-label applications such as prosthetic joint infections caused by MRSA or VRE, epidural abscesses, diabetic foot infections, and vertebral osteomyelitis [2]. With the increased use of daptomycin against resistant gram-positive organisms comes the risk of developing acute eosinophilic pneumonia, a rare, yet potentially, fatal adverse drug reaction [3].

Daptomycin-induced acute eosinophilic pneumonia (DEP) is the result of macrophage presentation of the drug or drug-hapten combination to helper T-cells, resulting in interleukin-5 release and subsequent eosinophil chemotaxis to the lungs. Although the exact mechanism of DEP is not known, it is thought to be related to the drug molecules binding to pulmonary surfactant and causing epithelial cell injury [4,5]. Clinically, acute eosinophilic pneumonia is characterized by fever, dyspnea, non-productive cough, and diffuse pulmonary infiltrates with crackles on auscultation, frequently progressing to acute hypoxic respiratory failure. Diagnostic criteria for DEP include recent daptomycin exposure, new infiltrates on computed tomography (CT), bronchoalveolar lavage (BAL) with greater than 25% eosinophils, and clinical improvement following withdrawal of the drug [5]. Prompt recognition and intervention in this acute inflammatory process can prevent further destruction of the lung parenchyma, lead to the reversal of damage, and allow for the resolution of symptoms.

## Case presentation

A 69-year-old female with a history of Hashimoto thyroiditis, right knee total knee arthroplasty, chronic obstructive pulmonary disease (COPD), and a recent diagnosis of mitral valve MRSA endocarditis on daptomycin presented to the emergency department (ED) with a history of right knee swelling for a few days. Three weeks prior, she was admitted to an outside hospital for confusion and was found to have mitral valve MRSA endocarditis and possible septic emboli to the brain. She was discharged with a plan to continue daptomycin treatment for a total of six weeks. Right knee pain, swelling, and warmth developed during this admission and persisted after discharge. Magnetic resonance imaging (MRI) of the right knee ordered by her primary care physician (PCP) showed a large joint effusion.

In the ED, vital signs and physical examination were notable for hypotension, tachycardia, right knee swelling, and bilateral rales. She was afebrile and breathing comfortably on room air. Initial labs revealed an elevated white blood cell count (WBC), eosinophilia, anemia, and thrombocytosis. C-reactive protein (CRP) was also elevated; however, lactic acid was normal. Initial lab values as well as their trends are presented in Table 1. Diagnostic arthrocentesis showed turbid fluid and was remarkable for the following elevated findings: 51,000 WBC/mm³, 9,000 red blood cells (RBCs)/mm³, and 97% neutrophils. Her chest X-ray showed basilar atelectasis and patchy infiltration in the lateral aspect of the right upper lobe. She was promptly treated with intravenous (IV) vancomycin, ceftriaxone, and fluids. With the concern of a right prosthetic joint infection in the setting of MRSA endocarditis, the patient underwent debridement and retention of the prosthesis, and intraoperative tissue was cultured for bacteria, fungi, and acid-fast bacilli.

**Table 1:** Trends in laboratory values. WBC: white blood cell count; AEC: absolute eosinophil count; CRP: C-reactive protein

	Day of admission	Day 3	Day 10	Day 15	Day 19	Day 22	Reference range
WBC	25.2	24.7	25.7	17.2	11.2	11.5	4–10 × 10³/µL
Hemoglobin	10.3	9.3	7.7	9	8.1	8.1	12–16 g/dL
AEC	1.26	0.94	1.8	0.17	0.34	0.56	0.03–0.46 × 10³/µL
Platelet count	536	562	756	880	653	574	150–450 × 10³/µL
Lactic acid	2		1.3				0.3–1.5 mmol/L
CRP	389		247	69			0–5 mg/L

**Figure 1:**
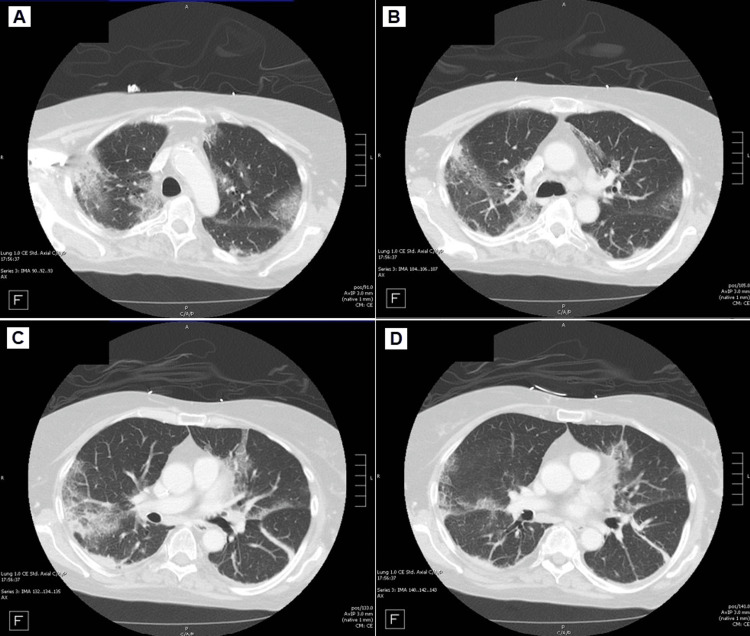
(A-D): Four panels of a CT scan of the chest on day five of admission showing upper lobe predominant interstitial thickening with ground-glass changes. CT: computed tomography

On admission, Infectious Disease (ID) recommended restarting IV daptomycin and discontinuing vancomycin and ceftriaxone. Because she was not a surgical candidate for mitral valve repair, the plan was to continue her previous regimen and treat her with daptomycin for six weeks. During her hospitalization, she became hypoxic, requiring 2 L of oxygen via a nasal cannula, and endorsed acute-on-chronic neck pain attributed to chronic cervical stenosis. She developed fevers on the third day of admission, necessitating the broadening of her antibiotic regimen to include IV piperacillin/tazobactam. A CT scan of the chest was ordered and showed interstitial thickening with upper lobe predominance and ground-glass changes (Figure 1), likely reflecting interstitial lung disease (ILD) without any consolidation. WBC and absolute eosinophil count continued to uptrend, as shown in Table 1. Piperacillin/tazobactam was discontinued after five days.

Due to increasing leukocytosis and persistent fevers, there was a concern for a brain abscess, especially given the previous septic emboli in the brain seen at the outside hospital. Other possible causes were considered, including vertebral osteomyelitis and persistent septic joint. The latter was considered low probability due to an unremarkable physical examination and lack of knee pain and swelling. Her history of COVID-19 and COPD was thought to be the cause of her hypoxia and non-productive cough, but upon review of her records from her PCP’s office from a few weeks prior, X-ray findings showed hyperinflation with no interstitial changes or other signs of ILD.

**Figure 2:**
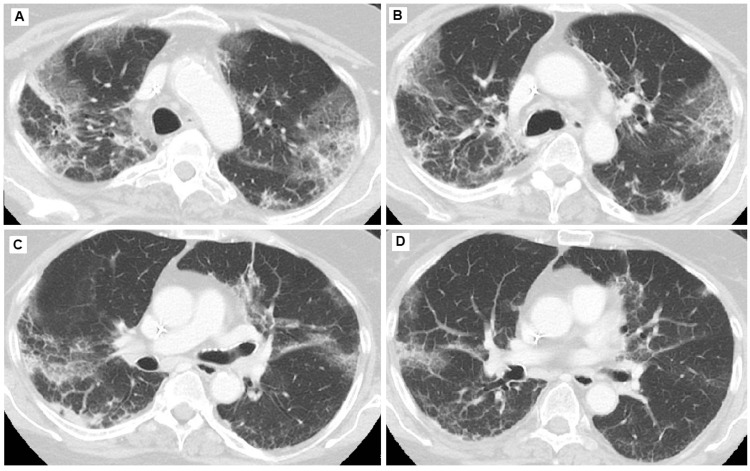
(A-D): CT scan of the chest on day 10 of admission showing interval increase in interstitial findings. CT: computed tomography

With persistent leukocytosis despite antibiotics, persistent peripheral eosinophilia, worsening interstitial lung infiltrates, and new oxygen requirement, there was a concern for daptomycin-induced eosinophilic pneumonia. Repeat CT chest showed worsening ground-glass opacities and septal thickening with a crazy-paving appearance (Figure 2).

**Figure 3:**
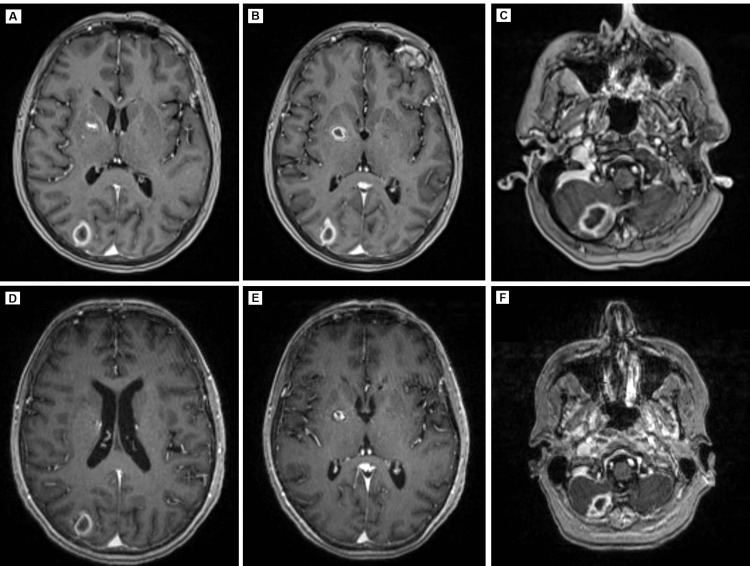
(A-C): MRI of the brain on day 12 of admission showing a 1.1 × 1.1 × 1.0 cm lesion in the right lentiform nucleus, a 1.9 × 1.3 × 1.7 cm lesion in the right occipital lobe, and a 2.5 × 1.4 × 1.6 cm lesion in the right cerebellum. (D-F): MRI of the brain on day 14 of admission showing a slight decrease in the size and no new lesions: a 9 × 7 mm lesion in the right lentiform nucleus, a 1.4 × 1.2 cm lesion in the right occipital lobe, and a 2.2 × 1.2 cm lesion in the right cerebellum. MRI: magnetic resonance imaging

Daptomycin was discontinued, IV vancomycin was started, and a bronchoscopy was requested. A CT of the head showed new ring-enhancing lesions in the same areas as the previously noted septic emboli (Figure 3, Panels A-C). An MRI of the brain confirmed the CT findings. Neurosurgery was consulted; medical management and serial brain MRIs were recommended as the abscesses were too small for neurosurgical intervention. On day 14, the patient underwent a bronchoscopy, and BAL was remarkable for the following elevated findings: 12% eosinophils, 31% neutrophils, and 11% mono/macrophages. Initial cultures were negative for *Aspergillus *and acid-fast bacilli but positive for *Candida albicans*, which was thought to be a colonizing organism.

**Figure 4:**
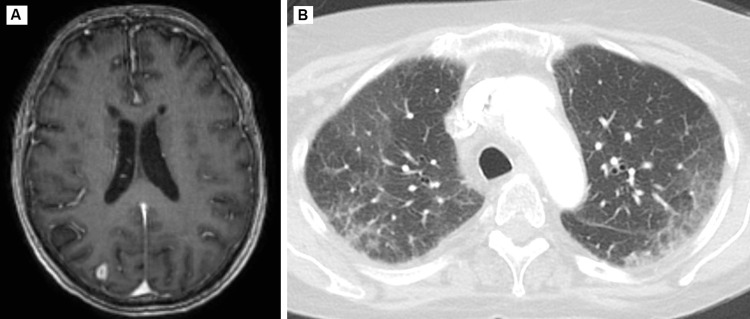
MRI of the brain (A) and CT scan of the chest (B) one month after discharge showing an overall decrease in lesion size and ground-glass opacities. MRI: magnetic resonance imaging; CT: computed tomography

The patient’s respiratory status improved three to four days after discontinuing daptomycin. Steroids were considered but not added to the treatment regimen due to her concurrent infection and risk of immunosuppression. Her dry cough resolved, she no longer had dyspnea on exertion, and she no longer required supplemental oxygen. On day 17, the patient reported feeling at baseline. Her second brain MRI noted no new ring-enhancing lesions and a slight decrease in the size of the abscesses compared to her previous study (Figure 3, Panels D-F). Her labs improved and her eosinophils normalized, as shown by the day 19 values in Table 1. The patient continued to improve clinically and was discharged after 22 days on IV vancomycin treatment for a total of eight weeks. A repeat MRI of the brain showed a continued decrease in lesion size (Figure 4A). A repeat chest CT after one month showed a decrease in ground-glass opacities (Figure 4B).

## Discussion

The following criteria characterize DEP: (1) >25% eosinophils on BAL, (2) fever, (3) clinical and radiographic evidence of pneumonia, and (4) disease onset and withdrawal immediately following drug initiation and cessation, respectively [5-7]. Our patient had 12% eosinophils on BAL, which may be explained by the fact that the sample was obtained three to four days after stopping daptomycin. Although the patient did not meet all the criteria, the diagnosis is still probable [8], with her pneumonia rapidly resolving following daptomycin withdrawal. The diagnosis is likely more difficult in the setting of the COVID-19 pandemic, given the prevalence of long-term sequelae and structural lung disease. To our knowledge, there are only two other case reports discussing DEP in the setting of COVID-19 [9,10]. Given our patient’s recent COVID-19 pneumonia and the complexity of her medical conditions, this diagnosis was challenging to identify among other confounding factors.

Long COVID-19 infection was considered given her history and persistent dry cough, but this was ruled out due to the absence of interstitial changes on chest X-ray four months prior and a negative COVID-19 nucleic acid amplification test. Likewise, *Aspergillus, Mycobacteria, Coccidioides*, and *Strongyloides* were eliminated by negative serologies and BAL culture. The prior chest X-ray, coupled with the absence of multisystemic involvement, made eosinophilic pneumonia with polyangiitis unlikely.

Hayes et al. first described DEP in a patient treated for endocarditis following a week of daptomycin therapy, and the phenomenon has since been increasingly reported [11]. Out of 196 patients with drug-induced eosinophilic pneumonia, daptomycin accounted for 32 cases [12]. Risk factors for DEP include male sex, older age, and prolonged daptomycin therapy [7]. Renal dysfunction is also common. Although our patient was older, she was female and did not have renal impairment.

Furthermore, the mean duration of daptomycin therapy before the onset of symptoms is three weeks [13]; our patient experienced DEP as early as 10 days after daptomycin treatment. Typical clinical findings include fever, dyspnea, malaise, and elevated CRP. Radiographic findings include patchy bilateral consolidations and ground-glass opacities, but these can vary [14]. Most cases received daptomycin for severe infection, most commonly endocarditis, prosthetic joint infection, osteomyelitis, or abscess. Withdrawal of daptomycin invariably leads to recovery, often within 72 hours. Our patient demonstrated a remarkable clinical course complicated by MRSA endocarditis, septic arthritis of a prosthesis, and septic brain emboli, making the prompt diagnosis and withdrawal of DEP critical in her recovery. Although DEP is increasingly reported, it remains a poorly understood phenomenon, and, to our knowledge, the current literature comprises only case reports or series. Given daptomycin’s primary indication for *Staphylococcus aureus* bacteremia or endocarditis, it is particularly pertinent for clinicians to identify iatrogenic DEP in a critically ill patient.

## Conclusions

When considering pneumonia as a diagnosis, it is important to have a low threshold of drug-induced etiologies. This was especially true in the case of this patient, as she was already on an antibiotic regimen at the initial presentation. Additionally, with a generous overlap in clinical presentation, this case adds to the limited body of literature surrounding acute eosinophilic pneumonia in the setting of COVID-19. Most importantly, due to the significant morbidity and mortality that this adverse drug reaction is associated with, this case highlights the importance of early recognition of DEP as well as early intervention.

## References

[1] Patel JJ, Antony A, Herrera M, Lipchik RJ (2014). Daptomycin-induced acute eosinophilic pneumonia. WMJ.

[2] Patel S, Saw S (2022). Daptomycin. StatPearls [Internet].

[3] Wu C, Li Z, Wang C, Deng Z (2023). Clinical characteristics, management, and outcome of eosinophilic pneumonia associated with daptomycin. Med Clin (Barc).

[4] Silverman JA, Mortin LI, Vanpraagh AD, Li T, Alder J (2005). Inhibition of daptomycin by pulmonary surfactant: in vitro modeling and clinical impact. J Infect Dis.

[5] Kumar S, Acosta-Sanchez I, Rajagopalan N (2018). Daptomycin-induced acute eosinophilic pneumonia. Cureus.

[6] Solomon J, Schwarz M (2006). Drug-, toxin-, and radiation therapy-induced eosinophilic pneumonia. Semin Respir Crit Care Med.

[7] Kim PW, Sorbello AF, Wassel RT, Pham TM, Tonning JM, Nambiar S (2012). Eosinophilic pneumonia in patients treated with daptomycin: review of the literature and US FDA adverse event reporting system reports. Drug Saf.

[8] Ahouansou N, Georges M, Beltramo G, Aswad N, Hassani Y, Bonniaud P (2021). Daptomycin-induced eosinophilic pneumonia: are there any risk factors?. Infect Dis Now.

[9] Watts A, Toquica Gahona CC, Raj K (2021). Multifocal pneumonia amidst the global COVID-19 pandemic: a case of daptomycin-induced eosinophilic pneumonia. Cureus.

[10] Valaiyapathi R, Wu MS, McGregor A (2022). Ground glass opacities are not always COVID-19: a case of acute eosinophilic pneumonitis caused by daptomycin. Lancet.

[11] Hayes D Jr, Anstead MI, Kuhn RJ (2007). Eosinophilic pneumonia induced by daptomycin. J Infect.

[12] Bartal C, Sagy I, Barski L (2018). Drug-induced eosinophilic pneumonia: a review of 196 case reports. Medicine (Baltimore).

[13] Uppal P, LaPlante KL, Gaitanis MM, Jankowich MD, Ward KE (2016). Daptomycin-induced eosinophilic pneumonia - a systematic review. Antimicrob Resist Infect Control.

[14] Higashi Y, Nakamura S, Tsuji Y (2018). Daptomycin-induced eosinophilic pneumonia and a review of the published literature. Intern Med.

